# Folate Receptor-Positive Gynecological Cancer Cells: In Vitro and In Vivo Characterization

**DOI:** 10.3390/ph10030072

**Published:** 2017-08-15

**Authors:** Klaudia Siwowska, Raffaella M. Schmid, Susan Cohrs, Roger Schibli, Cristina Müller

**Affiliations:** 1 Center for Radiopharmaceutical Sciences ETH-PSI-USZ, Paul Scherrer Institut, Villigen-PSI 5232, Switzerland; klaudia.siwowska@psi.ch (K.S.); raffaella.schmid@psi.ch (R.M.S.); susan.cohrs@psi.ch (S.C.); roger.schibli@psi.ch (R.S.); 2 Department of Chemistry and Applied Biosciences, ETH Zurich, Zurich 8093, Switzerland

**Keywords:** folate receptor, folic acid, ovarian cancer, cervical cancer, endometrial cancer, choriocarcinoma, KB, KB-V1, IGROV-1, SKOV-3, SKOV-3.ip

## Abstract

The folate receptor alpha (FR) is expressed in a variety of gynecological cancer types. It has been widely used for tumor targeting with folic acid conjugates of diagnostic and therapeutic probes. The cervical KB tumor cells have evolved as the standard model for preclinical investigations of folate-based (radio) conjugates. In this study, a panel of FR-expressing human cancer cell lines—including cervical (HeLa, KB, KB-V1), ovarian (IGROV-1, SKOV-3, SKOV-3.ip), choriocarcinoma (JAR, BeWo) and endometrial (EFE-184) tumor cells—was investigated in vitro and for their ability to grow as xenografts in mice. FR-expression levels were compared in vitro and in vivo and the cell lines were characterized by determination of the sensitivity towards commonly-used chemotherapeutics and the expression of two additional, relevant tumor markers, HER2 and L1-CAM. It was found that, besides KB cells, its multiresistant KB-V1 subclone as well as the ovarian cancer cell lines, IGROV-1 and SKOV-3.ip, could be used as potentially more relevant preclinical models. They would allow addressing specific questions such as the therapeutic efficacy of FR-targeting agents in tumor (mouse) models of multi-resistance and in mouse models of metastases formation.

## 1. Introduction

The folate receptor alpha (FR) has emerged as an interesting tumor target due to its overexpression in a variety of tumor types, including several gynecological cancers of epithelial origin [1,2,3]. The occurrence of FRs in normal tissue is limited, with kidneys being the most important site of physiological FR-expression [4,5]. Due to favorable FR-targeting properties, the vitamin folic acid has been investigated extensively as a ligand to deliver attached diagnostic and therapeutic payloads for imaging and therapy of FR-expressing cancer [6]. This targeting concept is based on the accessibility of folic acid for chemical derivatization allowing the conjugation of even bulky entities without losing FR-binding affinity [7]. 

Tumor targeting using radionuclides conjugated to folic acid was shown to be effective for nuclear imaging using single photon emission computed tomography (SPECT) and positron emission tomography (PET) in numerous pre-clinical experiments as well as in the clinics [8,9,10]. Moreover, folate conjugates of fluorescent probes have been developed for intraoperative imaging of ovarian tumors allowing more radical cytoreductive surgery in patients [11]. With regard to FR-targeted tumor therapy, many approaches have been reported in the literature, among those the most promising being the coupling of folic acid with anticancer drugs [12,13,14,15,16,17,18]. A number of otherwise highly toxic agents have been used in conjunction with folic acid to allow specific accumulation in FR-expressing tumor cells for cancer therapy in clinical trials [19,20,21,22,23,24].

The choice of an appropriate tumor model to investigate the concept of FR-targeting in preclinical settings remains challenging. In this regard, KB tumor cells are most often used and, hence, they are considered as the “gold standard” for this purpose [17]. In the past, this cell line was believed to be a human nasopharyngeal epidermal carcinoma cell line [25,26,27,28], however, later it became obvious that the KB cell line was established via contamination by HeLa cells, a cervical cancer cell line [29,30]. KB cells are readily used for any investigation with regard to FR-targeting due to their high FR-expression level as well as fast growth and general ease of culturing. The question arises, however, whether this model is the most appropriate for preclinical research.

As no comprehensive study exists, in which different FR-positive tumor cell lines are investigated and compared, the in vitro and in vivo characterization of such cell lines appeared important. An overview in this regard would not only facilitate the design of future research in the field, but allow for the selection of an appropriate tumor cell type when testing combination therapies with chemotherapeutics or other treatment modalities. Moreover, it may allow a better understanding of the differences between in vitro and in vivo models as well as the challenges which may occur when translating in vitro results to the in vivo situation.

There is a number of human gynecological cancer cell lines which are known, or mentioned in the literature, to express the FR [27]. These include cell lines of cervical and ovarian cancer, as well as choriocarcinoma and endometrial tumor types. The use of the cervical adenocarcinoma cells is most common in the field, due to the very high FR-expression level in KB cells [31,32,33,34]. KB cells are a subclone of HeLa cells, which is the first human epithelial cancer cell line established in culture and probably the best-known cell line in past and current research [35]. The multidrug-resistant KB-V1 cell line has been derived from KB cells by culturing them with increasing amounts of vinblastine [36]. The multi-drug resistance (MDR)-1 gene of KB-V1 cells encodes P-glycoprotein responsible for decreased intracellular accumulation of anticancer agents, such as vinca alkaloids, doxorubicin, daunorubicin, paclitaxel, actinomycin D or etoposide [37,38,39].

Strong attention should be drawn towards ovarian cancer, since this cancer type is known to express the FR with highest frequency (~90% of the cases) in patients [3]. The human ovarian adenocarcinoma cell line, IGROV-1, was proposed as a model for human ovarian cancer in 1985 [40], and later employed for FR-targeting studies [41]. SKOV-3 is another ovarian cancer cell line found to express the FR [42,43,44]. Yu et al. reported on the development of SKOV-3.ip cells, generated by isolating tumor cells from the ascites of a mouse injected with SKOV-3 intraperitoneally [43]. The SKOV-3.ip subclone was characterized with a higher level of c-erbB-2/neu expression, as well as more aggressive peritoneal carcinomatosis. It was proposed as a more relevant model of ovarian cancer since multiple metastasis-like tumors grow in the abdomen when the cells are injected into the peritoneum of mice [43]. Two choriocarcinoma cell lines, JAR and BeWo, were mentioned in the literature to be FR-positive [45,46,47]. These cell lines were used as models for malignant tumors of the trophoblast [48,49,50]. Finally, there was an indication that endometrial EFE-184 tumor cells also express the FR (oral communication). This cell line may be of interest to be used as a model of endometrial cancer, since clinically, this cancer type is also reported to express the FR with high frequency (~90%) [3].

In the present study, the aim was to characterize these FR-positive cell lines in vitro and in vivo. The relative FR-expression level and the ability to bind folate radioconjugates were investigated in cultured cells. The most promising cancer cell lines were tested with regard to their ability to grow in mice. FR-expression was evaluated on tissue sections of xenografts using in vitro autoradiography and immunohistochemistry. Finally, the in vivo targeting was demonstrated using a recently-developed folate radioconjugate (^177^Lu-cm10 [51]) in the four tumor mouse models that revealed the greatest potential to be used for FR-targeting research.

## 2. Results and Discussion

### 2.1. In Vitro Culturing of FR-Expressing Cancer Cell Lines

**Figure 1:**
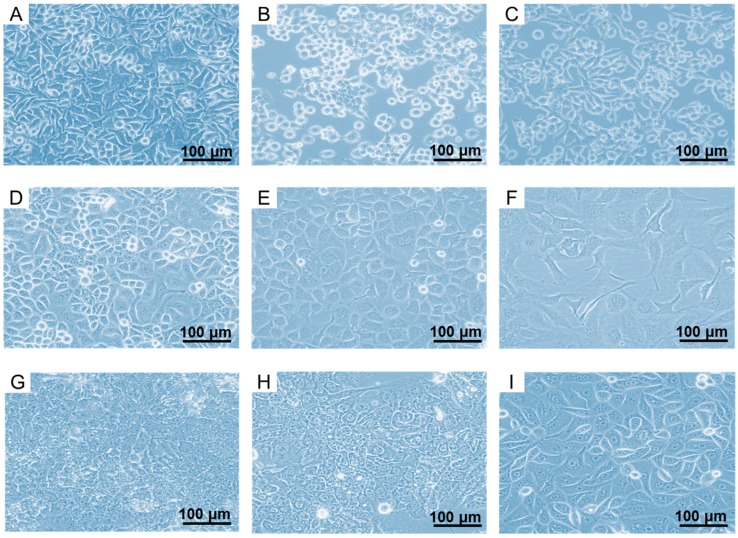
Microscopic images of (**A**) HeLa cells; (**B**) KB cells and (**C**) KB-V1 cells; (**D**) IGROV-1 cells; (**E**) SKOV-3 cells and (**F**) SKOV-3.ip cells; (**G**) JAR cells; (**H**) BeWo cells and (**I**) EFE-184 cells. Magnification 20×.

All cell lines were grown in folate-deficient RPMI medium (FFRPMI) with fetal calf serum (FCS) as the only source of folate. The expression of the FR allowed these cell lines to grow at very low folate concentrations when most FR-negative cell lines would not survive. Different morphology and confluency levels were observed for each cancer cell line even among the same tumor type as shown in the microscopic images (Figure 1). There was a tendency of faster growth of KB-V1 and SKOV-3.ip cells as compared to the parental cells, KB and SKOV-3, respectively. Most of the cell lines showed tight adherence to the culture flasks, however, KB-V1 and BeWo cells were more challenging to culture and for being used in experiments as they showed weak adherence, requiring surface-coated cell culture flasks and well-plates.

### 2.2. Determination of FR-Expression Levels of Cells Cultured In Vitro

**Figure 2:**
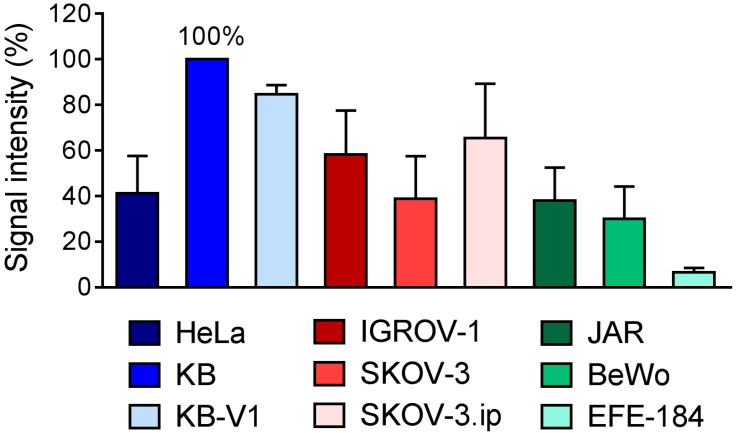
Quantification of signal intensity obtained from western blot for FR-expression in cervical, ovarian, choriocarcinoma and endometrial cancer cell lines. The value obtained for KB cells was set as 100% and the percentage of the signals of the other cell lines was calculated for each single western blot (*n* = 5–6) and expressed as the average ± standard deviation.

Western Blot technique was used to determine relative FR-expression levels in all cancer cell lines (Figure 2, Supplementary Materials Figure S1). Among the tested cervical cancer cell lines, KB cells revealed the most prominent FR-expression, followed by KB-V1 and HeLa cells, the latter showing clearly reduced levels despite being frequently used in FR-targeting research [52,53]. In ovarian cancer cells FR-expression levels were almost identical in IGROV-1 and SKOV-3.ip tumor cells. In SKOV-3 cells, however, FR-expression was lower and comparable to the expression level in HeLa cells. In the choriocarcinoma cells, JAR and BeWo, the FR was detected as well, but at lower levels. The western blot signal obtained with EFE-184 cells was very weak, indicating low FR-expression levels. Comparison of FR-expression in all investigated cancer cell lines, independent of the tumor type, revealed the following sequence: KB > KB-V1 > SKOV-3.ip > IGROV-1 > HeLa ≈ SKOV-3 ≈ JAR > BeWo > EFE-184. Based on these results, IGROV-1 or SKOV-3.ip ovarian cancer cell lines appeared most promising after KB and KB-V1 cells to be used for FR-targeting.

**Figure 3:**
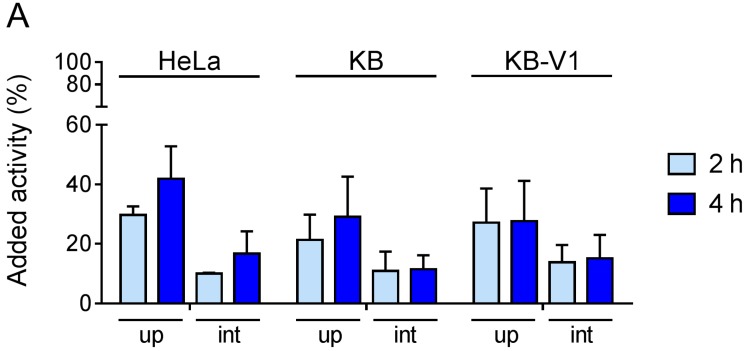

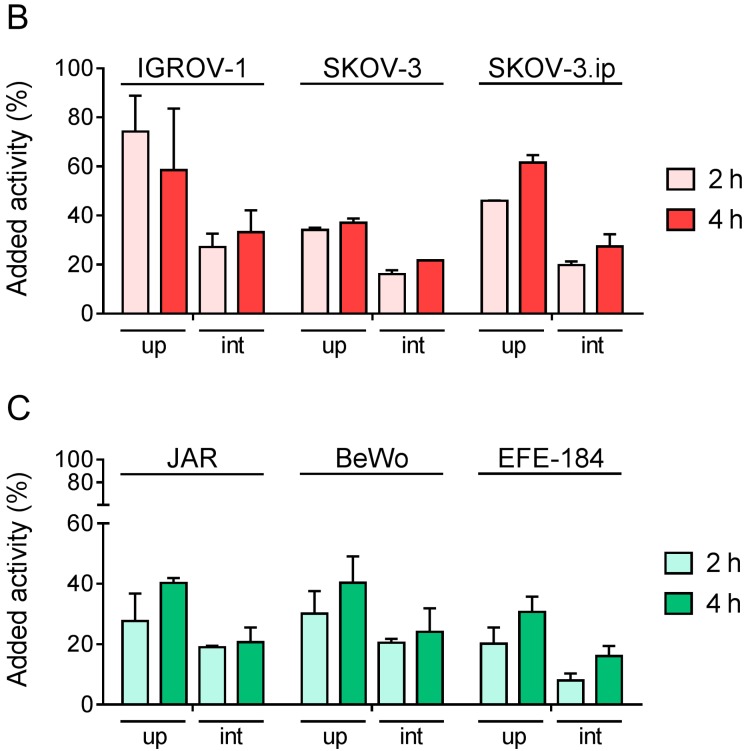
Total uptake (up) and internalization (int) of ^177^Lu-folate in (**A**) cervical cancer cells; (**B**) ovarian cancer cells; (**C**) choriocarcinoma cells and endometrial cancer cell.

As next step, the ability of these cell lines to actively accumulate folate conjugates via FR-mediated uptake was investigated in vitro using a radiolabeled folate conjugate (^177^Lu-cm10, [51]) previously developed in our group (Figure 3). In cervical cancer cells, the total uptake of the radiofolate was in the range of 21–42% of added activity whereas about 12% and 15% were internalized after 2 h and 4 h incubation, respectively (Figure 3A). IGROV-1 and SKOV-3.ip cells showed high radiofolate uptake reaching 60–70% of added activity. Interestingly, these ovarian cancer cells showed higher radiofolate uptake than KB cells, despite lower expression of FRs. These findings are in agreement with literature reports where it is stated that the FR-expression level is not proportional to the uptake of folates [7]. The uptake in SKOV-3 cells was more comparable to the uptake in cervical cancer cell lines. JAR and BeWo cells showed equally high uptake and internalization comparable to HeLa, KB, KB-V1 and SKOV-3 cells. Slightly reduced values were found in the case of EFE-184 cells in comparison to JAR and BeWo. Generally, the internalized fraction was about one third up to half of the total uptake (referring to the sum of surface-bound and internalized fraction) of radiofolate. In addition, experiments with excess folic acid to block FRs prior to the addition of the radiofolate resulted in reduced uptake and internalization to less than 1% which unambiguously indicated FR-specific binding of the radiofolate (Figure 3).

### 2.3. Tumor Cell Characterization beyond FR-Expression

#### 2.3.1. Expression of L1-Cell Adhesion Molecule

As a further characterization of these cancer cell lines we determined the expression levels of L1-cell adhesion molecule (L1-CAM), a frequently expressed antigen in ovarian cancer known to correlate with the aggressiveness of cancer (Supplementary Materials Figure S2A) [54,55,56]. L1-CAM was detected in all three cervical cancer cell lines. In ovarian cancer cells, SKOV-3 and SKOV-3.ip cells, showed significant expression of L1-CAM whereas in IGROV-1 cells the expression level appeared to be lower. L1-CAM-expression may be of relevance, as it was shown that downregulation of L1-CAM in IGROV-1 cells led to decreased cell proliferation [57]. In line with this observation, the treatment of SKOV-3.ip cells with an antibody against L1-CAM showed significantly decreased proliferation [58]. Interestingly, choriocarcinoma cells did not show any expression of L1-CAM, however, high expression levels were found in EFE-184 cells. Since L1-CAM was previously associated with a poor prognosis in endometrial cancer [59,60], it is likely that EFE-184 cells are representative for an aggressive cancer cell type.

#### 2.3.2. Expression of Human Epidermal Growth Factor Receptor-2

Human epidermal growth factor receptor 2 (HER2) is an epidermal growth factor receptor 2, overexpressed in 10–15% of breast cancers and associated with a poor prognosis [61]. It is a common marker of breast cancer, however, also found in ovarian cancer, with the incidence indicated between 8% and 66% depending on the literature [62]. Although the significance of HER2 is clearly established in breast cancer, its role is not as clear in ovarian cancer. Treatment of ovarian cancer with trastuzumab, an anti-HER2 antibody resulted in an overall response rate of only ~7% in patients with HER2-positive ovarian cancer [63], whereas in breast cancer patients the overall response rate was 15–18% [64]. The detailed investigation of the role of HER2 in ovarian cancer and other non-breast cancers is currently an important topic of research. Therefore, we set out to investigate the cell lines with regard to HER2-expression (Supplementary Materials Figure S2B).

Data on HER2-expression in cervical cancer is not consistently reported in the literature [65], however, in our study expression of HER2 was not detected in cervical cancer cell lines. Among the ovarian cell lines tested in this study, HER2-expression was found in SKOV-3 and SKOV-3.ip tumor cells, in line with the literature [66]. However, other than in previous studies, we did not find much difference in HER2-expression levels among these cell lines. HER2 was also reported to be expressed at a moderate level in IGROV-1 cells [67], however, in the present study it was not detected in this cell line. In choriocarcinoma, the HER2-expression was reported to be associated with an invasive phenotype [68]. While JAR cells did not show expression of HER2, a signal was detected for BeWo cells, potentially indicating a more invasive phenotype of this choriocarcinoma cell line.

#### 2.3.3. Sensitivity towards Chemotherapeutics

The characterization of the investigated cancer cell lines was additionally addressed by determination of their sensitivity towards the treatment with commonly used chemotherapeutics (Table 1). 5-Fluorouracil (5-FU), gemcitabine (GEM) and pemetrexed (PMX) are antimetabolites which are employed or tested as radiosensitizing agents for application in radio-oncology [69,70,71]. 5-FU reduced cell viability when applied in the micromolar range. KB cells showed reduced sensitivity to 5-FU as compared to KB-V1 (Table 1), despite the latter being characterized as multi-drug resistant (MDR) [36]. These findings are in agreement with previous studies suggesting that the MDR-1 gene expression does not cause resistance against 5-FU [72]. IGROV-1 and SKOV-3.ip demonstrated values in the same range, whereas the SKOV-3 cells were less sensitive towards 5-FU. BeWo cells were 2-fold more sensitive than JAR and EFE-184 cells. The IC_50_ values for all investigated cell lines treated with GEM were in the nanomolar range. From the most sensitive to the most resistant cancer cell line IC_50_, values varied over two orders of magnitude. KB cells were less sensitive than HeLa cells and KB-V1 cells were the most sensitive among cervical cancer cells. These findings are in line with the literature, where it was reported that multidrug resistant cells are more sensitive toward gemcitabine treatment than their parental cell lines [73]. Among ovarian cancer cell lines, SKOV-3.ip was the least sensitive. JAR cells were less sensitive than BeWo cells, which revealed to be most sensitive among all tested cell lines. Finally, EFE-184 cells showed an IC_50_ value which was in the same range as for ovarian cancer cells.

Among all three antimetabolites, PMX was most effective in reducing tumor cell viability resulting in IC_50_ values in the low nanomolar range with only slight variability among different cell lines. Cervical cancer cell lines were more sensitive than ovarian and choriocarcinoma cell lines and EFE-184 cells emerged as the most resistant, as ~50% viable cells were found even with very high concentrations of PMX.

Cisplatin (CIS), doxorubicin (DOX) and paclitaxel (PCX) are important chemotherapeutics since they are used as a standard therapy of ovarian cancer [74,75]. All investigated cell lines showed similar sensitivity to CIS in the low micromolar range and even the more aggressive versions, KB-V1 and SKOV-3.ip did not show any resistance against this chemotherapeutic agent. In the case of DOX and PCX, the multidrug resistant cell line, KB-V1 revealed to be more resistant than KB or HeLa cell lines as expected and previously shown [76]. Among ovarian cancer cells, SKOV-3.ip cells were much more resistant to DOX as compared to SKOV-3 and IGROV-1 cells. This can be considered as another indication that SKOV-3.ip cells are an aggressive subtype of ovarian cancer cells. On the other hand, no difference in sensitivity was determined towards PCX among ovarian cancer cells as previously reported [77]. BeWo cells reacted again more sensitive to the treatment with DOX and PCX as compared to JAR cells. EFE-184 cells proved again to be resistant, demonstrated by much higher IC_50_ values after treatment with DOX and PCX as compared to choriocarcinoma cells.

Sensitivity of FR-positive cell lines to the commonly used chemotherapeutics is of crucial interest for the investigation of FR-targeted therapeutics, as these novel therapy concepts might be a solution in chemoresistant tumors. Cell lines generally considered as invasive or aggressive, such as KB-V1 and SKOV-3.ip, overexpress the FR at very high levels and may be more susceptible to the FR-targeted therapies.

### 2.4. Gynecologic Tumor Xenograft Mouse Models

Based on the in vitro results, cervical and ovarian cancer cell lines appeared more promising to be used in vivo than JAR, BeWo and EFE-184 cells. These FR-expressing cancer cell lines were, therefore, investigated with regard to their potential to grow as xenografts in nude mice. Since it was reported that HeLa, KB and KB-V1 cells can be grown in CD-1 nude mice [27], this strain was used for in vivo experiments. The KB tumor mouse model is the best established and has been used for a large number of in vivo investigations of radiofolates in the past [18,51,78,79,80,81]. KB tumors are characterized with a fast growth and a solid, firm structure. In this study, it was confirmed that KB-V1 tumor cells also grow fast in nude mice when inoculated subcutaneously. It appeared that KB-V1 tumors were better vascularized compared to KB tumors as was visible by a more reddish color of KB-V1 xenografts. The growth of HeLa cells in CD-1 nude mice was very slow and in some cases, the xenografts started to shrink after about 2–3 weeks and disappeared completely. The ovarian cancer cell lines were also grown as subcutaneous xenografts in CD-1 nude mice. IGROV-1 and SKOV-3.ip reached a tumor size suitable for in vivo experiments within about 2 weeks as reported previously [82]. On the other hand, SKOV-3 cells grew very slowly and the resulting tumor xenografts remained small even several weeks after tumor cell inoculation. PC-3 cells, used as FR-negative control, were also grown in CD-1 nude mice.

### 2.5. FR-Expression Levels in Tumor Xenografts

#### 2.5.1. Determination of FR-Expression Using Autoradiography

**Figure 4:**
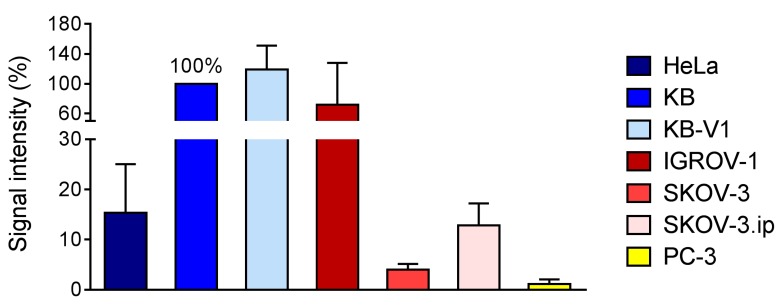
Quantification of in vitro autoradiography results in tumor tissues. Values obtained for KB cell line were set as 100% and compared with the other tissues.

FR-expression levels were compared in tumor xenografts of cervical and ovarian carcinoma cells as well as in PC-3 xenograft using the technique of in vivo autoradiography. Based on the obtained signal, it was revealed that FR-expression in KB and KB-V1 tumors was comparable, but much lower in the case of HeLa tumor tissue (Figure 4, Supplementary Materials Figure S3). Among the ovarian tumor tissue sections, the most intense signal was obtained for the IGROV-1 tumors, whereas the signal intensity of SKOV-3.ip tumor tissue was in the range of HeLa tumor sections, indicating similar FR-expression levels. Only the signal of SKOV-3 tumor tissue was much lower. Incubation of the tumor tissue sections with excess folic acid blocked the receptors and reduced the signal to background levels which confirmed FR-specific binding of the radiofolate. The FR-negative PC-3 tumor sections served as negative control revealing a signal of ~1% (Figure 4, Supplementary Materials Figure S3). In general, these findings were in line with those of western blot analysis with the exception being SKOV-3 and SKOV-3.ip cells, which showed high FR-expression in vitro, but when grown as xenografts in mice, FR-expression appeared to be significantly reduced.

#### 2.5.2. Determination of FR-Expression Using Immunohistochemistry

**Figure 5:**
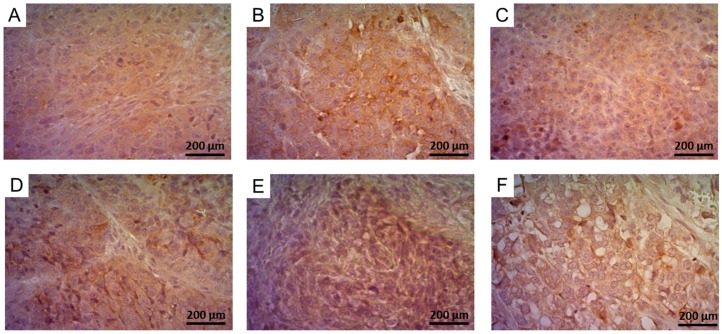
Immunohistochemistry results showing FR-expression in (**A**) HeLa; (**B**) KB; (**C**) KB-V1; (**D**) IGROV-1; (**E**) SKOV-3 and (**F**) SKOV-3.ip tumors. Tissue images are shown in magnification 40×.

FR-expression levels in tumor xenografts were additionally investigated by immunohistochemistry and a semi-quantitative analysis was performed (Figure 5, Supplementary Materials, Figures S4 and S5). Similar to the result of the autoradiography, HeLa tumor tissue showed a 10% reduced staining, indicating lower FR-expression levels as compared to KB and KB-V1 tumor tissue which showed an intense staining signal. In comparison to the FR-staining of KB tumor tissue the signal was reduced by 6% in the case of IGROV-1 tumor tissue which was in agreement with the in vitro autoradiography results (Figure 4). The signal obtained for the SKOV-3 tumor tissue was slightly higher than the signal obtained for SKOV-3.ip tumor tissue (12% and 20% lower signal than for KB tumor tissue, respectively). This data was not in line with the autoradiography results possibly due to the fact that the tissue texture of SKOV-3.ip tumors was different than the tissue of the other tumors. The analysis of the results revealed significantly higher values of all tumor tissue sections as compared to PC-3 tumor tissue, which served as a negative control. Absence of tissue staining was obtained in negative control experiments performed on tissue sections treated without the primary antibody (Supplementary Materials Figure S4). 

### 2.6. Biodistribution Experiments

**Figure 6:**
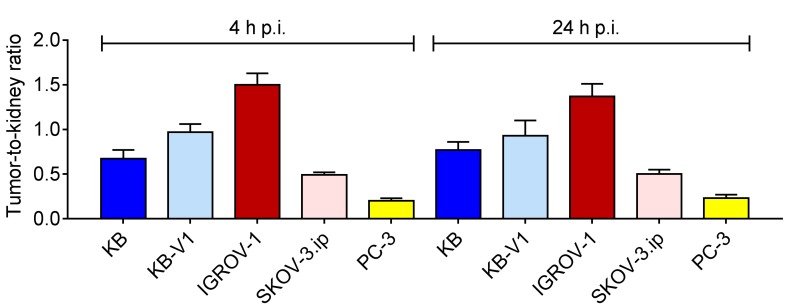
Tumor-to-kidney ratios of accumulated radioactivity in tumor-bearing mice 4 h and 24 h after injection of the radiofolate. The tumor-to-kidney ratios of all groups of mice bearing FR-positive tumor types (KB, KB-V1 or IGROV-1, respectively) were significantly different (*p* < 0.05) than the tumor-to-kidney ratio in PC-3 tumor-bearing mice. An exception was the tumor-to-kidney ratio of SKOV-3.ip tumor-bearing mice which was significantly different (*p* < 0.05) from the ratios in PC-3 tumor-bearing mice only at 4 h p.i. but not (*p* > 0.05) at 24 h p.i. of the radiofolate.

The tumor growth was investigated starting from day 4 after inoculation of tumor cells by measuring tumor xenografts every second day (Supplementary Material, Figure S6). Comparison of accumulated radiofolate in different tumor types was performed based on tumor-to-kidney ratios in order to standardize the results to kidney uptake which should be equal for each mouse independent of the xenograft type (Figure 6). The analysis revealed the highest accumulation of activity in IGROV-1 tumors at both investigated time points after injection. A possible explanation for these findings may be the fact, that IGROV-1 tumors were smaller (116 ± 70 mm^3^ at day 14 after inoculation) in comparison to other FR-positive tumors and possibly better vascularized (Supplementary Materials Figure S6). KB tumor xenografts grew very fast (189 ± 73 mm^3^ at day 12 after inoculation) and appeared to be less vascularized. This was confirmed by SPECT/CT images where it was seen that the activity was mainly accumulated in the outer rim of the tumor but not homogenously distributed within the whole tumor xenografts (Supplementary Material Figure S7). KB-V1 tumor cells were also found to grow fast even though the tumors were smaller (123 ± 86 mm^3^ at day 12 after cell inoculation). Nevertheless, both KB and KB-V1 tumors accumulated high amounts of activity which was in line with high levels of FR-expression in these tumor types as demonstrated by autoradiography and immunohistochemistry experiments (Figure 4 and Figure 5, Supplementary Material Figures S3–S5). SKOV-3.ip tumors reached a tumor volume (134 ± 48 mm^3^ at day 12 after cell inoculation) and were in the same range as KB-V1 tumors. Tumor-to-kidney ratios of accumulated activity in SKOV-3.ip tumor-bearing mice were higher as compared to PC-3 tumor-bearing mice which served as a negative control, however, the differences were minimal and not significant at the 24 h time point. Thus, it may be that in the case of SKOV-3.ip tumors, the accumulation of the radiofolate was mostly due to the blood flow rather than as a consequence of FR-specific uptake.

In agreement with this analysis, it was found that the absolute tumor uptake 24 h after injection of the radiofolate was highest for IGROV-1 tumor xenografts (~34% IA/g) followed by KB (~22% IA/g), KB-V1 (~17% IA/g) and SKOV-3.ip tumors (~13% IA/g). A clearly reduced accumulation of activity was found in PC-3 tumors (~6% IA/g) at the same time point (Supplementary Materials Tables S1 and S2).

## 3. Conclusions

A crucial aspect for the development of FR-targeted imaging and therapeutic agents is to use a suitable model for preclinical investigations. Until now, KB tumor cells have been the “gold standard” for in vitro and in vivo FR-targeting research, however, other tumor models may be of interest in order to take the diversity of naturally occurring cancers into account. In this study, we investigated tumor cells of cervical, ovarian and endometrial origin as well as choriocarcinoma cells. KB, KB-V1, IGROV-1 and SKOV-3.ip cells revealed to be appropriate for in vitro experiments and could be efficiently grown in mice allowing tumor targeting in vivo. KB cells were confirmed to be a very useful model for FR-targeting research. KB-V1 tumor cells are a valid alternative, which would be of particular interest when multiresistance should be investigated. IGROV-1 tumor cells are favorable when the research refers to ovarian cancer, however, these cells appeared to be more challenging than KB tumor cells in terms of reproducible in vivo growth. Finally, the SKOV-3.ip tumor cell line would be attractive for the performance of research on mouse models with metastases-like tumors. It has to be kept in mind, however, that the SKOV-3.ip cell line expresses the FR at lower levels than it is the case for IGROV-1 tumor cells.

Using these additional tumor cell lines can enable investigation of folate-based therapeutics in more detail as they would allow addressing specific questions such as their therapeutic efficacy in tumor (mouse) models of multi-resistance and in models of metastases formation. 

## 4. Materials and Methods

### 4.1. General

Pemetrexed (PMX, Alimta^TM^, Eli Lilly, Indianapolis, IN, USA), doxorubicin (DOX, doxorubicin hydrochloride, Sigma-Aldrich, St. Louis, MO, USA) and gemcitabine (GEM, Gemzar^TM^, Eli Lilly) were obtained as lyophilized powders and dissolved in sterile NaCl 0.9% according to the instructions of the manufacturer. 5-Fluorouracil (5-FU, Fluorouracil-Teva^TM^, Teva Pharma AG, Basel, Switzerland), cisplatin (CIS, Actavis, Actavis Switzerland AG, Regensdorf, Switzerland) and paclitaxel (PCX, Paclitaxel Sandoz^TM^, Sandoz Pharmaceuticals AG, Rotkreuz, Switzerland) were obtained as solutions for injection. The solutions were diluted in sterile NaCl 0.9% to obtain the required concentration. The folate conjugate cm10 (referred to as folate herein) [51], was kindly provided by Merck & Cie, Schaffhausen, Switzerland. No carrier added lutetium-177 (^177^Lu) was obtained from Isotope Technologies Garching (ITG) GmbH, Munich, Germany.

### 4.2. Preparation of ^177^Lu-Folate

The ^177^Lu-folate was prepared under standard labeling conditions as previously reported [18,51]. Quality control of the prepared ^177^Lu-folate was performed via reversed-phase high performance liquid chromatography as previously reported [51]. The radiochemical purity of ^177^Lu-folate was always >97%.

### 4.3. Cell Lines and Cell Culture

HeLa cells (cervical carcinoma cell line, ACC-57), KB cells (cervical carcinoma cell line, subclone of HeLa cells, ACC-136), KB-V1 cells (cervical carcinoma cell line, multi-drug resistant (MDR) subclone of KB cells, ACC-14), BeWo cells (choriocarcinoma cell line, ACC-458), JAR cells (choriocarcinoma cell line, ACC-462) EFE-184 cells (endometrial carcinoma, ACC-230) and PC-3 cells (human prostate cancer cell line, FR-negative, ACC-465) were purchased from the German Collection of Microorganisms and Cell Cultures (DSMZ, Braunschweig, Germany). SKOV-3 cells (ovarian adenocarcinoma cell line, ECACC Cat-N° 91091004) were purchased from Culture Collections, Public Health England, Salisbury, United Kingdom. SKOV-3.ip, an ovarian carcinoma cell line established from ascites of a nude mouse developed after intraperitoneal injection of SKOV-3 cells [43], were kindly provided by Dr. Ilse Novak (Paul Scherrer Institut, Villigen, Switzerland). IGROV-1 cells (human ovarian carcinoma cell line) were a kind gift from Dr. Gerrit Jansen (Department of Rheumatology, Free University Medical Center, Amsterdam, The Netherlands). Cervical (HeLa, KB, KB-V1), ovarian (SKOV-3, SKOV-3.ip, IGROV-1), choriocarcinoma (JAR, BeWo) and endometrial carcinoma cells (EFE-184) were cultured in folate-deficient RPMI medium (FFRPMI, Cell Culture Technologies GmbH, Gravesano, Switzerland) supplemented with 10% FCS, l-glutamine and antibiotics. PC-3 cells were cultured in standard RPMI 1640 medium supplemented with 10% FCS, l-glutamine and antibiotics. Routine cell culture was performed twice a week using trypsin-EDTA (0.25%, Gibco) for detachment of the cells. Standard cell culture flasks were used for all cells except BeWo and KB-V1 which were cultured in cell culture flasks with a hydrophilic surface, obtained after microwaving process (Corning). All experiments with these two cell lines were performed in poly-l-lysine coated well-plates.

### 4.4. Cell Internalization Experiments

Materials and methods of cell internalization experiments are reported in Supplementary Materials. Graphs were prepared using GraphPad Prism software (version 7.0, La Jolla, CA, USA). Data represents the average of two to four different experiments.

### 4.5. Western Blot

Western blot was performed with cell lysates (~40 μg protein) using anti-FR antibody (Abcam, Cambridge, UK, mouse Ab, ab3361, 1:500), anti-L1CAM antibody (chCE-7, IgG1-subtype chimeric monoclonal human antibody [83], 5 μg/mL) and anti-HER2/erbB-2 antibody (Cell Signaling Technology, Danvers, MA, USA, rabbit Ab, #2165, 1:1000). The detailed procedure is described in Supplementary Materials.

Western Blot signal was quantified using ImageJ software (version 1.51k, NIH, Rockville, MD, USA). Region of interest (ROI) was chosen manually, based on the largest band in the blot. The same ROI was applied in all remaining rows, with the protein band in the middle of the ROI frame. The mean signal of each ROI was standardized to the signal of KB cells, which was set as 100%. The result is an average of percentage from five to six different experiments. 

### 4.6. In Vitro Autoradiography

Tumor xenografts collected from mice were fixed in embedding material (Cryo-M-Bed, Bright) and frozen at −80 °C. Tumor tissue sections of 5–10 μm thickness were prepared using a cryostat (Bright OTF Cryostat, OTF/AS-001/MR/V/304/X, Huntingdon, UK). Data represents the average of three different experiments. The detailed procedure of the autoradiography experiments is described in Supplementary Materials.

### 4.7. Immunohistochemistry

Tumor xenografts were embedded in paraffin and cut into 5 μm-thick sections using a manual rotary microtome (Leica RM2235, Leica Biosystems, Wetzlar, Germany). Removal of paraffin was performed with xylene, followed by rehydration with decreasing ethanol concentrations. Citrate buffer (10 mM trisodium citrate/0.05% Tween buffer, pH 6) was used for antigen retrieval in 95 °C for 30 min. Endogenous peroxidase was blocked by incubation of the slides in a solution of 3% H_2_O_2_. Unspecific binding was prevented by incubation of the slides in 10% FCS for 60 min. Avidin/biotin blocking kit (SP-2001, Vector Laboratories, Burlingame, CA, USA) was used according to the manufacturer’s protocol. The primary anti-FR antibody (Abcam, ab67422) was added in a concentration of 0.5 μg/100 μL and slides were incubated overnight at 4 °C. Slides were incubated with biotinylated secondary antibody (Abcam, ab97049, 1:200) for 30 min followed by addition of the Avidin-Biotin Complex kit (ABC Reagent kit, Elite, Vectastain, Vector Laboratories) and incubation for 30 min. DAB peroxidase substrate kit (Vector Laboratories, SK-4100) was used for the development of the signal and hematoxylin (Novolink™, Leica Biosystems) for counterstaining. The sections were treated with increasing concentrations of ethanol before treatment with xylene for fixation. Pictures were obtained using a light microscope (Axio Lab.A1, Zeiss, Oberkochen, Germany).

### 4.8. Animal Experiments

In vivo experiments were conducted in accordance with the Swiss law of animal protection. Athymic nude mice (Crl:CD-1-Foxn1 nu, referred herein as CD-1 nude) were purchased from Charles River Laboratories (Sulzfeld, Germany). Animals were inoculated with a suspension of the tumor cells (5–7 × 10^6^ tumor cells in 100 μL PBS) subcutaneously on the right shoulder or both shoulders and 5 × 10^6^ tumor cells in the case of a biodistribution study. All animals were fed with a folate-deficient rodent diet (ssniff Spezialdiäten GmbH, Soest, Germany).

### 4.9. Biodistribution Experiments

Biodistribution studies were performed 12–14 days after inoculation of the tumor cells when the tumor xenografts reached a volume between 63 mm^3^ and 189 mm^3^. ^177^Lu-folate conjugate (3 MBq, 0.5 nmol per mouse) was injected in a volume of 100 μL PBS into a lateral tail vein. The animals were sacrificed at 4 h (*n* = 4) and 24 h (*n* = 4) after administration of the radioconjugate. Blood and selected tissues and organs were collected, weighed, and radioactivity was measured using a γ-counter (Perkin Elmer, Wallac Wizard 1480, Waltham, MA, USA). The results were listed as a percentage of the injected radioactivity per gram of tissue mass (% IA/g), using counts of a defined volume of the original injection solution measured at the same time resulting in decay-corrected values. The significance of the data was determined using a one-way analysis of variance (ANOVA) with Bonferroni’s multiple comparison post-test (GraphPad Prism Software, version 7.00). A *p* value of <0.05 was considered statistically significant.

## Figures and Tables

**Table 1:** IC_50_ values of cells treated with 5-fluorouracil (5-FU), gemcitabine (GEM), pemetrexed (PMX), cisplatin (CIS), doxorubicin (DOX) and paclitaxel (PCX).

Cancer Type	Cell Line	5-FU IC_50_ (μM)	GEM IC_50_ (nM)	PMX IC_50_ (nM)	CIS IC_50_ (μM)	DOX IC_50_ (nM)	PCX IC_50_ (nM)
Cervical	HeLa	16.2	56.4	8.9 **	2.8	36.2	9.5
	KB	28.6 *	120	4.5 **	1.9	15.4	2.7
	KB-V1	9.1	12.8	5.3	0.3	211	597
Ovarian	IGROV-1	2.0	11.8	41.8	0.8	23.9	5.0
	SKOV-3	8.0 **	20.8	14.9 *	5.6	96.7 **	4.0
	SKOV-3.ip	3.1	45.0 *	6.4 *	1.9	449 **	9.6
Choriocarcinoma	JAR	8.2	30.6	33.8	0.9	9.8	3.1
	BeWo	4.2	1.2	6.4	0.4	2.2	1.6
Endometrial	EFE-184	9.7 *	15.8	n.d.	1.6	390	90.9

* At the highest applied concentration ~20% cells were still viable; ** at the highest applied concentration ~30% cells were still viable. In one case cells could not be killed entirely, even with very high amounts of the drug. In this case, the IC_50_ could not be determined (n.d.).

## Supplementary Materials

The following figures are available online at www.mdpi.com/1424-8247/10/3/72/s1, Figure S1: Western blot analysis of the folate receptor (FR) in different gynecologic cancer cell lines, Figure S2: Western blot analysis of L1-CAM (A) and HER2 (B) in different gynecologic cancer cell lines, Figure S3: Autoradiography results, Figure S4: Immunohistochemistry results of FR-expression in FR-expressing tumors, Figure S5: Semi-quantitative analysis of FR expression levels determined by immunohistochemistry, Figure S6: Tumor growth in mice inoculated with different cell lines, Figure S7: SPECT/CT images of tumor-bearing mice injected with ^177^Lu-folate, Tables S1 and S2: Results of biodistribution experiment.
