# Supplementary material for: Folate Receptor-Positive Gynecological Cancer Cells: In Vitro and In Vivo Characterization
Source: Pharmaceuticals (Basel). 2017 Aug 15;10(3):72. doi: 10.3390/ph10030072 (PMC5620616; doi:10.3390/ph10030072)
Supplement: Supplementary file 1 [file pharmaceuticals-10-00072-s001.pdf]

Supplementary Materials: Folate Receptor-Positive Gynecological Cancer Cells: In Vitro and In Vivo Characterization

Klaudia Siwowska [1], Raffaella M. Schmid [1], Susan Cohrs [1], Roger Schibli [1,2] and Cristina Müller [1,*]

[1] Center for Radiopharmaceutical Sciences ETH-PSI-USZ, Paul Scherrer Institut, Villigen-PSI 5232, Switzerland; klaudia.siwowska@psi.ch (K.S.); raffaella.schmid@psi.ch (R.M.S.), susan.cohrs@psi.ch (S.C.), roger.schibli@psi.ch (R.S.)

[2] Department of Chemistry and Applied Biosciences, ETH Zurich, Zurich 8093, Switzerland; roger.schibli@pharma.ethz.ch (R.S.)

* Correspondence: cristina.mueller@psi.ch; Tel.: +41-56-310-44-54

1. Cell Internalization Experiments

Experimental Procedure

Cells were seeded in 12-well plates (~0.5–1.0 × 10^6 cells in 2 ml folate-free (FFRPMI) medium/well) allowing cell adhesion and growth overnight at 37 °C. After removal of the supernatant, cells were washed once with PBS prior to the addition of FFRPMI medium without supplements (975 µL/well). ^{177}Lu-folate (25 µL, ~38 kBq, ~1.5 pmol) was added to each well. In some cases, cells were incubated with an excess folic acid (100 µM) to block FRs on the surface of the cancer cells. After incubation of the well plates for 2 h or 4 h at 37 °C, the cells were washed twice with ice-cold PBS to determine total uptake of ^{177}Lu-folate. In order to assess the fraction of internalized ^{177}Lu-folate, the cells were additionally washed with a stripping buffer (aqueous solution of 0.1 M acetic acid and 0.15 M NaCl, pH 3 [1]), to release FR-bound ^{177}Lu-folate from the cell surface. Cell samples were lysed by addition of NaOH (1 M, 1 mL) to each well. The cell suspensions were then transferred to tubes for measuring in a γ-counter (Perkin Elmer, Wallac Wizard 1480). After vortexing of the cell suspension, the concentration of the proteins was determined using a Micro BCA Protein Assay Kit (Pierce, Thermo Scientific) in order to standardize the measured radioactivity to the average content of protein in a single well. The results were expressed as percentage of total added radioactivity.

2. Western Blot Analysis

2.1. Purpose

The purpose of this investigation was the determination and comparison of tumor antigens including the folate receptor alpha (FR), the L1-cell adhesion molecule (L1-CAM) and the human epidermal growth factor receptor 2 (HER2) in gynecologic cancer cells cultured in vitro.

2.2. Experimental Procedure

Cell lysates (~40 µg protein) were separated by sodium dodecyl sulfate polyacrylamide gel electrophoresis (SDS-PAGE). Proteins were transferred to a polyvinylidene fluoride membrane and the binding sites were blocked with 2% bovine serum albumin (BSA) for FR or non-fat dry milk for HER2 and L1-CAM in Tris-buffered saline containing 0.05% Tween™ 20 (TBST). Overnight incubation at 4 °C was performed using anti-FR antibody (Abcam, mouse Ab, ab3361, 1:500), anti-L1CAM antibody (chCE-7, IgG1-subtype chimeric monoclonal human antibody [2], 5 µg/mL) and anti-HER2/erbB-2 antibody (Cell Signaling, rabbit Ab, #2165, 1:1000). A secondary IgG antibody (anti-mouse from Cell Signaling #70765, 1:5000, anti-rabbit from Cell Signaling, #7074, 1:5000, or anti-human from Promega, W4031, 1:10000) was used together with a chemiluminescent substrate (SuperSignal™ West Pico Chemiluminescent Substrate, Thermo Scientific) to develop the signal.

2.3. Results and Conclusion

FR-expression was detected at high levels in KB, KB-V1, IGROV-1 and SKOV-3.ip cell lines (Figure S1). HeLa and SKOV-3 cell lines showed a less intense signal, however, the FR-expression was still relatively high. JAR and BeWo cells expressed the FR at a moderate level, whereas EFE-184 cells showed only very weak signal indicating very low FR-expression.

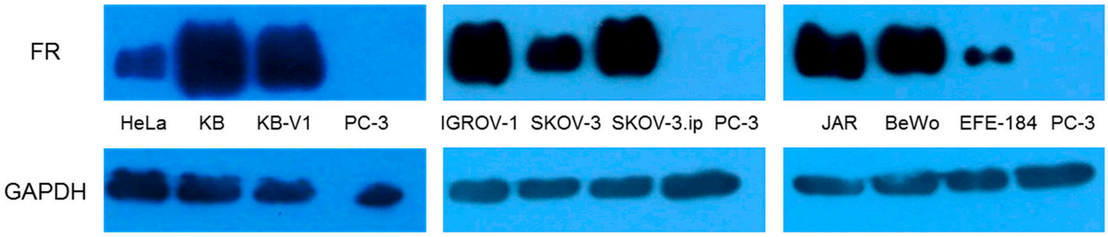

Figure S1. Western blot analysis of the folate receptor (FR) in different gynecologic cancer cell lines.

Cervical cancer cell lines showed high levels of L1-CAM expression (Figure S2A). Among the three cell lines tested in this study, KB cells showed the weakest signal. Among ovarian cancer cell lines SKOV-3.ip and SKOV-3 cells showed a more intense signal than IGROV-1 cells indicating higher L1-CAM expression. JAR and BeWo cells did not show any signal, indicating that they do not express L1-CAM, however, EFE-184 endometrial cancer cells showed high levels of the protein (Figure S2A). No expression of L1-CAM was observed in the negative control, fibrosarcoma cell line, HT1080 [3]. Double band was presumably detected due to the cleavage and different glycosylation levels of the protein when expressed in vitro [4].

Only three of the investigated cell lines showed HER2 expression on western blot analysis (Figure S2B). These were SKOV-3.ip and SKOV-3 cells as well as BeWo (Figure S2B). In this case, PC-3 cell lysate was used as a negative control [5].

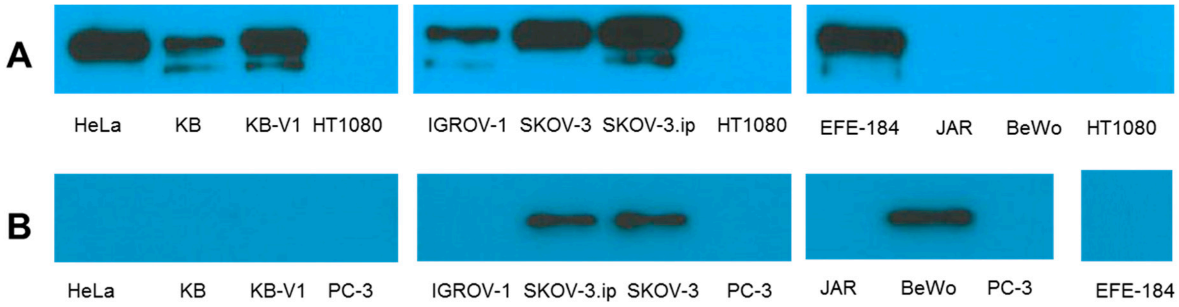

Figure S2. Western blot analysis of (**A**) L1-CAM and (**B**) HER2 in different gynecologic cancer cell lines.

3. In Vitro Autoradiography

3.1. Purpose

In order to get an indication about the FR-expression in vivo, autoradiography studies were performed on frozen tumor sections of xenografts grown in nude mice.

3.2. Experimental Procedure

Slides were thawed and incubated in Tris-buffer (167 mM Tris-HCl, 5 mM $MgCl_2$) containing 0.25% BSA for 10 min at room temperature. ^{177}Lu-folate prepared at a specific activity of 50 MBq/nmol was diluted in Tris-buffer containing 1% BSA (1 MBq/mL) and added on the slides (100 μL) followed by incubation for 1 h. Blockade of FRs was performed using the same solution containing excess folic acid (100 μM). Activity was removed and slides were washed twice for 5 min with cold Tris-buffer containing 0.25% BSA. Next, the Tris-buffer without BSA was added for 5 min. The last step was

repeated with tap water. After drying the slides, images were obtained and quantified using a storage phosphor system (Cyclone Plus, Perkin Elmer) and OptiQuant Software (version 5.0, Bright Instrument Co Ltd, Perkin Elmer™). The signal intensity obtained for KB tumor sections, after incubation with the radiofolate, was set to 100% while the signal intensity of all other tissue sections was calculated accordingly.

3.3. Results and Conclusion

FR-expression was investigated on tumor sections of HeLa, KB, KB-V1, IGROV-1, SKOV-3, SKOV-3.ip and PC-3 cells. In cervical cancer tissue, high binding of ^{177}Lu-folate was observed for KB and KB-V1 tumors, whereas for HeLa tumors the signal was clearly reduced (Figure S3A, main Manuscript Figure 4). The most prominent uptake in ovarian tumor xenografts was observed in tissue sections of IGROV-1 and SKOV-3.ip tumor xenografts while SKOV-3 tumor showed a weaker signal (Figure S3C). In FR-negative PC-3 tumor sections, there was no binding of the radiofolate. Tumor samples were also incubated with a mixture of ^{177}Lu-folate and excess folic acid, to block FRs and therewith confirm FR-specific binding of the radiofolate (Figure S3B/D).

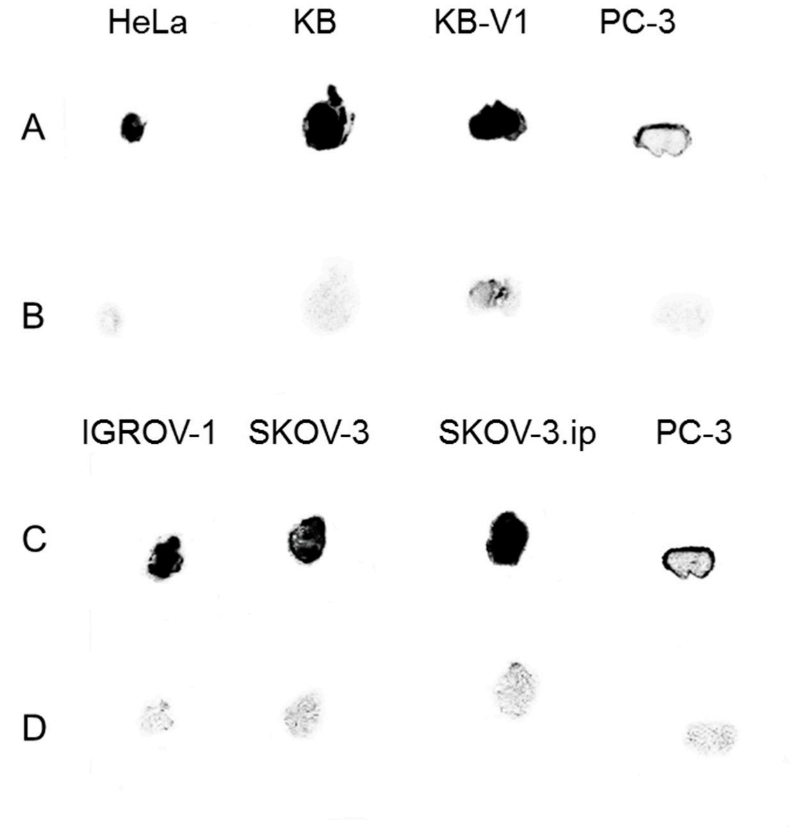

Figure S3. Autoradiography results showing accumulation of the radiofolate in (**A/B**) cervical tumor xenografts and (**C/D**) ovarian tumor xenografts. (**A/C**) Tumor sections exposed to ^{177}Lu-folate alone and (**B/D**) tumor sections exposed to a mixture of ^{177}Lu-folate and excess folic acid in order to block FRs.

4. Immunohistochemistry

4.1. Purpose

In order to get an indication about the FR-expression in vivo, immunohistochemistry was performed on paraffin tumor sections of xenografts grown in nude mice.

4.2. Experimental Procedure

The experimental procedure of the immunohistochemistry is reported in the main manuscript. Semi-quantitative analysis was performed using ImageJ program (version 1.51k) and Image Deconvolution plugin. The scale of 8-bit greyscale DAB images was converted to a minimum value of 0 and a maximum value of 255. The results were presented as the mean grey value of six images from the same tissue sample for each tumor type and the coefficients of variation were calculated by dividing the standard deviation by the mean value (CV = SD/mean). The significance of the data was determined using a one-way analysis of variance (ANOVA) with Bonferroni's multiple comparison post-test (GraphPad Prism Software, version 7.00). A p-value of <0.05 was considered statistically significant.

4.3. Results

The results of the immunohistochemistry (IHC) experiments are reported in the main manuscript (Figure 5). Absence of tissue staining was obtained in negative control samples treated without the primary antibody (Figure S4).

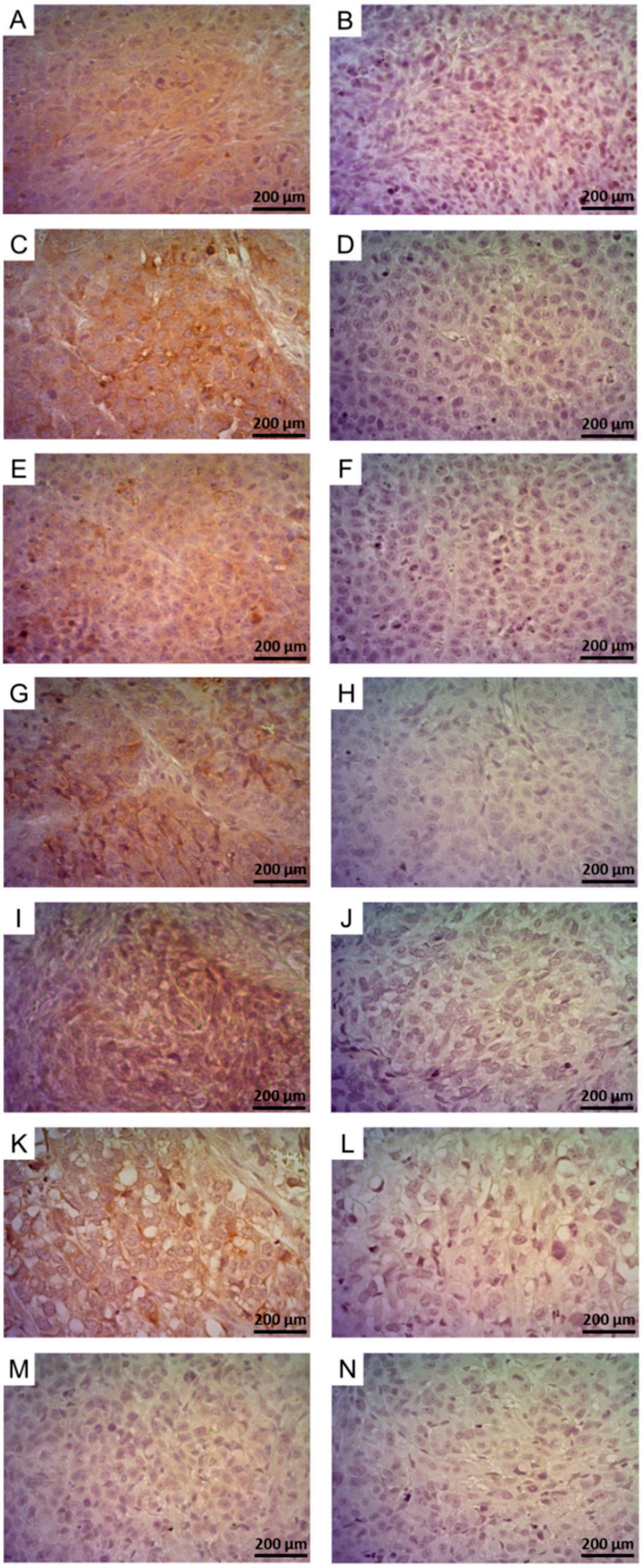

Figure S4. FR-expression in (**A/B**) HeLa, (**C/D**) KB, (**E/F**) KB-V1, (**G/H**) IGROV-1, (**I/J**) SKOV-3, (**K/L**) SKOV-3.ip and (**M/N**) PC-3 tumors. Tissue sections are shown in magnification 40 x. Images **A**, **C**, **E**, **G**, **I**, **K** and **M** demonstrate the stained tissue while **B**, **D**, **F**, **H**, **J**, **L** and **N** show the negative control staining without the primary antibody.

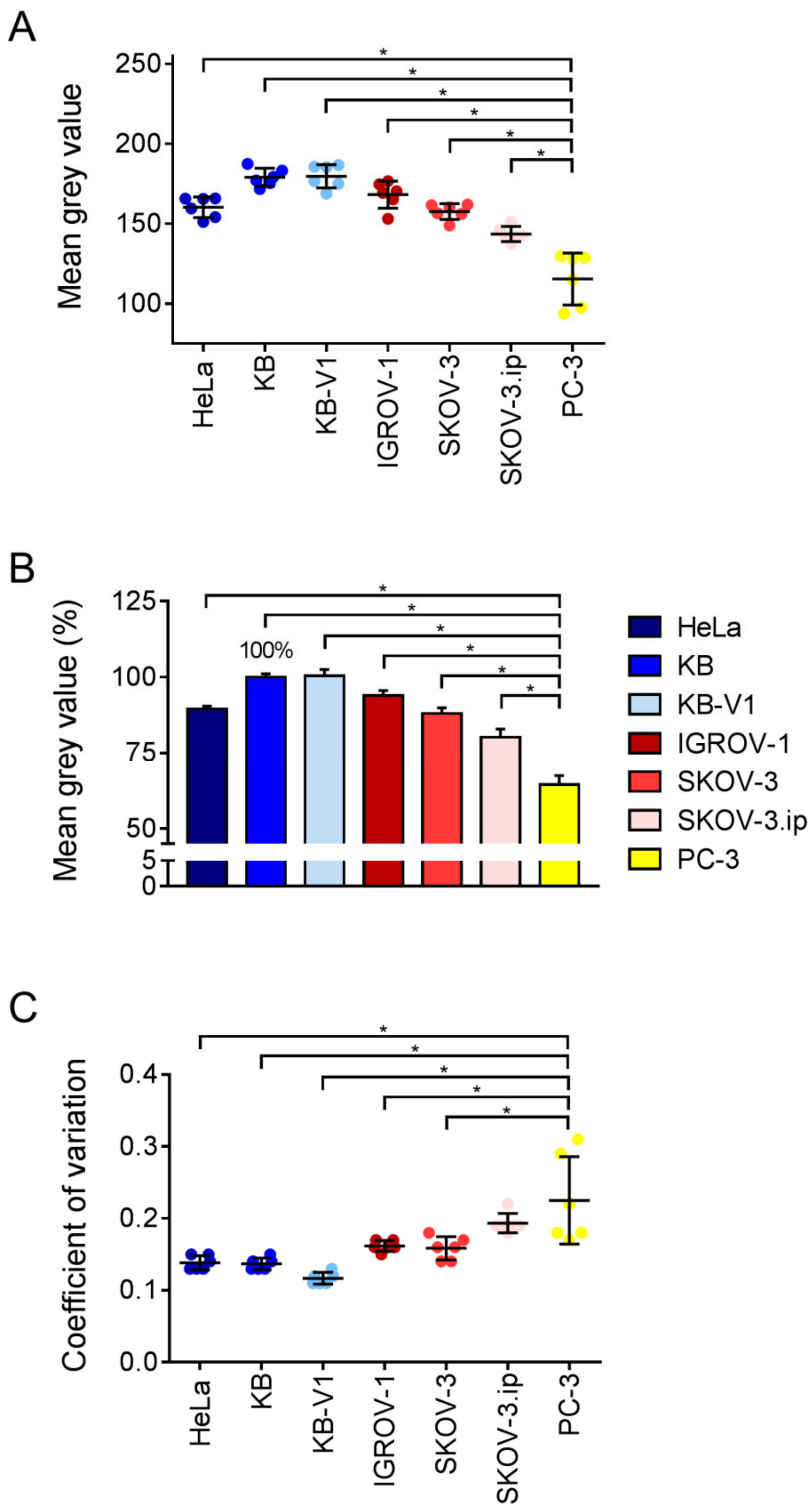

Figure S5. Semi-quantitative analysis of FR-expression levels determined by IHC experiments in HeLa, KB, KB-V1, IGROV-1, SKOV-3 and SKOV-3.ip tumor tissue. (**A**) Average of mean grey value of DAB-staining, (**B**) average of mean grey value expressed in percent of the value obtained for KB tumor tissue which was set as 100%. (**C**) Coefficient of variation was calculated for all the samples and plotted on the graph.

5. Monitoring of Tumor Growth in Mice

5.1. Experimental Procedure

CD-1 nude mice were inoculated subcutaneously with 5 x 10^6 tumor cells at both shoulders. The tumor growth was monitored for each cell type in 8 mice over a period of ~2 weeks. The tumors were measured every second day and the tumor volume (V) was determined according to the equation $[V = 0.5 \times (L \times W^2)]$, where L is the longest axis and W is the perpendicular axis to L [6].

5.2. Results and Conclusion

To compare the growth of each tumor type in mice, the same number of cells of each tumor cell type was subcutaneously inoculated. The monitoring of the tumor size revealed a relatively fast and consistent tumor growth of KB, KB-V1 tumor xenografts reaching a volume of ~189 mm^3 and ~123 mm^3, respectively, at day 12 after inoculation (Figgure S6). SKOV-3.ip tumors were also growing fast and reached a volume of ~134 mm^3 after 12 days, when the biodistribution experiment was performed. Due to the slower tumor growth for IGROV-1 and PC-3 tumors, mice were used for biodistribution studies only at day 14 after tumor cell inoculation when the tumor size reached a volume of 116 mm^3 and 63 mm^3, respectively.

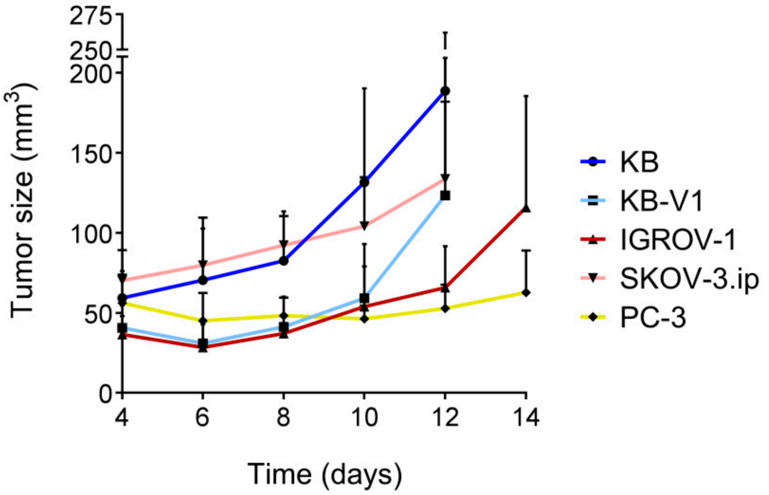

Figure S6. Tumor growth in mice inoculated with KB, KB-V1, IGROV-1, SKOV-3.ip and PC-3 tumors.

6. Biodistribution Studies

6.1. Experimental Procedure

The experimental procedure of the biodistribution studies is reported in the main manuscript.

6.2. Results

The results of the biodistribution studies are shown in Table S1 and Table S2.

Table S1. Biodistribution 4 h after injection of ^{177}Lu-folate in mice bearing KB, KB-V1, IGROV-1, SKOV-3.ip and PC-3 tumors.

	KB	KB-V1	IGROV-1	SKOV-3.ip	PC-3
Blood	4.24 ± 1.31	3.75 ± 0.81	4.19 ± 0.58	5.31 ± 1.11	5.34 ± 0.32
Spleen	1.00 ± 0.31	0.79 ± 0.13	0.94 ± 0.08	1.21 ± 0.39	1.19 ± 0.14
Kidneys	31.6 ± 4.64	24.1 ± 4.46	24.8 ± 1.10	26.6 ± 2.55	31.0 ± 2.26
Liver	2.94 ± 0.54	2.36 ± 0.38	2.49 ± 0.62	2.77 ± 0.55	3.16 ± 0.06
Salivary glands	6.45 ± 0.93	5.50 ± 0.63	6.53 ± 0.58	6.26 ± 0.88	7.62 ± 0.30
Muscle	1.38 ± 0.56	1.41 ± 0.45	1.54 ± 0.21	1.56 ± 0.56	1.72 ± 0.49
Bone	1.28 ± 0.32	1.21 ± 0.08	1.39 ± 0.11	1.63 ± 0.17	1.81 ± 0.40
Tumor	21.3 ± 4.84	23.1 ± 2.49	37.0 ± 3.82	13.1 ± 1.59	6.14 ± 1.25
Tu-to-blood	5.13 ± 0.69	6.29 ± 0.75	8.92 ± 0.99	2.51 ± 0.31	1.15 ± 0.17
Tu-to-liver	7.47 ± 2.44	9.92 ± 1.02	15.5 ± 3.26	4.80 ± 0.44	1.95 ± 0.38
Tu-to-kidney	0.67 ± 0.10	0.97 ± 0.09	1.50 ± 0.13	0.49 ± 0.03	0.20 ± 0.03

Table S2. Biodistribution 24 h after injection of ^{177}Lu-folate in mice bearing KB, KB-V1, IGROV-1, SKOV-3.ip and PC-3 tumors.

	KB	KB-V1	IGROV-1	SKOV-3.ip	PC-3
Blood	0.55 ± 0.05	0.79 ± 0.08	0.92 ± 0.08	1.39 ± 0.11	1.00 ± 0.02
Spleen	0.52 ± 0.05	0.47 ± 0.06	0.62 ± 0.16	0.84 ± 0.17	0.81 ± 0.26
Kidneys	27.9 ± 5.44	18.3 ± 3.07	25.3 ± 3.96	24.9 ± 1.92	27.1 ± 0.55
Liver	1.78 ± 0.22	1.34 ± 0.18	1.55 ± 0.10	1.56 ± 0.16	1.84 ± 0.37
Salivary glands	3.78 ± 0.30	3.12 ± 0.85	3.50 ± 0.79	4.46 ± 1.12	3.83 ± 0.32
Muscle	1.54 ± 0.63	1.10 ± 0.19	1.09 ± 0.41	1.32 ± 0.18	1.16 ± 0.38
Bone	0.84 ± 0.06	0.76 ± 0.19	0.88 ± 0.12	1.13 ± 0.08	1.04 ± 0.12
Tumor	21.5 ± 4.92	16.7 ± 2.73	34.4 ± 4.61	12.5 ± 1.02	6.13 ± 1.18
Tu-to-blood	38.8 ± 5.30	21.2 ± 3.26	37.3 ± 4.60	9.04 ± 0.80	6.13 ± 1.14
Tu-to-liver	12.3 ± 3.38	12.5 ± 1.48	22.3 ± 2.98	8.05 ± 0.49	3.42 ± 0.84
Tu-to-kidney	0.77 ± 0.09	0.93 ± 0.17	1.37 ± 0.14	0.50 ± 0.05	0.23 ± 0.04

7. In Vivo SPECT/CT Imaging

7.1. Experimental Procedure

Imaging studies were performed using a four-head, multiplexing, multipinhole small-animal SPECT/CT camera (NanoSPECT/CT™, Mediso Medical Imaging Systems, Budapest, Hungary). ^{177}Lu-folate (~20-25 MBq corresp. ~0.8-1.0 nmol) was injected into the lateral tail vein of tumor-bearing mice about 2-3 weeks after tumor cell inoculation. SPECT scans were performed 4 h after injection of ^{177}Lu-folate, followed by CT scans. The images were acquired using Nucline Software (version 1.02, Mediso Ltd., Budapest, Hungary). The real-time CT reconstruction used a cone-beam filtered backprojection. The reconstruction of SPECT data was performed using HiSPECT software (version 1.4.3049, Scivis GmbH, Göttingen, Germany). Images were prepared using the VivoQuant post-processing software (version 2.10, inviCRO Imaging Services and Software, Boston, U.S.). A Gauss post-reconstruction filter (FWHM = 1 mm) was applied for the presentation of the SPECT images and the scale was adjusted to the same kidney uptake in each mouse to allow visual comparison of radioactivity uptake in tumors.

7.2. Results and Conclusion

SPECT/CT imaging experiments were performed with mice bearing KB, KB-V1, IGROV-1, SKOV-3.ip and PC-3 tumors, 4 h after injection of ^{177}Lu-folate (Figure S7). As previously shown in FR-targeting studies [7], KB tumor xenografts showed high accumulation of radioactivity allowing a clear

visualization of the tumor xenograft. Radioactivity detected in the kidneys can be ascribed to the expression of the FR in the proximal tubule cells that leads to specific binding of radiofolates which are excreted through the kidneys [8]. Somewhat increased uptake of radioactivity was observed in KB-V1 tumor xenografts as compared to KB tumors, presumably due to a better vascularization of KB-V1 tumors allowing the folate radioconjugate to penetrate the tumor tissue better. In line with the biodistribution data, IGROV-1 tumor xenografts showed also a higher uptake than KB tumors. The uptake in SKOV-3.ip was visible, however, the signal was very weak. In agreement with the autoradiography and immunohistochemistry results, SKOV-3.ip expressed the FR at lower levels in vivo as compared to KB, KB-V1 and IGROV-1 tumor xenografts. As expected, radiofolate accumulation was not visible in FR-negative PC-3 tumor xenografts of mice used as controls and only kidneys could be visualized (Figure S7).

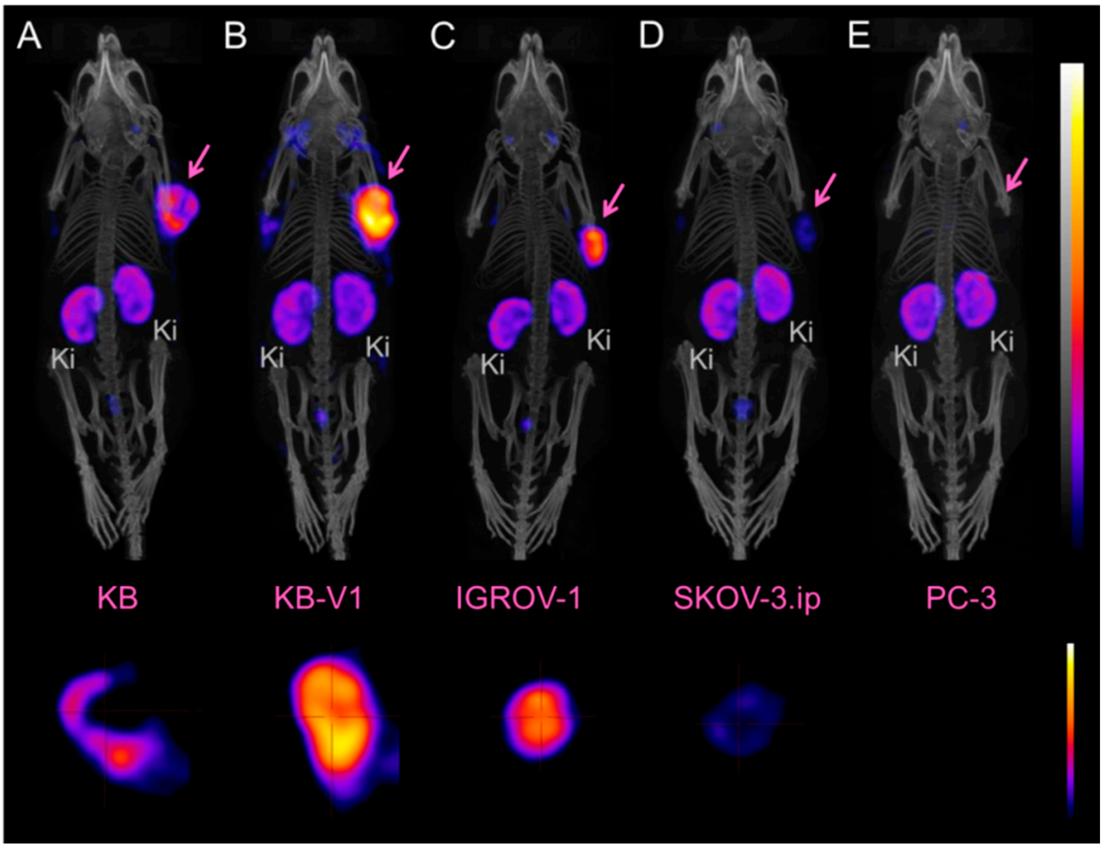

Figure S7. Whole-body SPECT/CT images presented as maximal intensity projections (upper panel) and sagittal sections of zoomed tumor xenografts (lower panel): (**A**) KB, (**B**) KB-V1, (**C**) IGROV-1, (**D**) SKOV-3.ip and (**E**) negative control, PC-3 tumor-bearing mice 4 h after injection of ^{177}Lu-folate. Tumors are indicated with pink arrows; Ki = kidney.

References

1. Ladino, C.A.; Chari, R.V.J.; Bourret, L.A.; Kedersha, N.L.; Goldmacher, V.S. Folate-maytansinoids: Target-selective drugs of low molecular weight. *Int. J. Cancer* **1997**, *73*, 859–864.
2. Grünberg, J.; Knogler, K.; Waibel, R.; Novak-Hofer, I. High-yield production of recombinant antibody fragments in HEK-293 cells using sodium butyrate. *Biotechniques* **2003**, *34*, 968–972.
3. Hauser, S.; Bickel, L.; Weinspach, D.; Gerg, M.; Schäfer, M.K.; Pfeifer, M.; Hazin, J.; Schelter, F.; Weidle, U.H.; Ramser, J., *et al.* Full-length L1CAM and not its Δ2Δ27 splice variant promotes metastasis through induction of gelatinase expression. *PLoS One* **2011**, *6*, e18989.
4. Schäfer, M.K.; Altevogt, P. L1CAM malfunction in the nervous system and human carcinomas. *Cell Mol. Life Sci.* **2010**, *67*, 2425–2437.

5. Carrion-Salip, D.; Panosa, C.; Menendez, J.A.; Puig, T.; Oliveras, G.; Pandiella, A.; De Llorens, R.; Massaguer, A. Androgen-independent prostate cancer cells circumvent EGFR inhibition by overexpression of alternative her receptors and ligands. *Int. J. Oncol.* **2012**, *41*, 1128–1138.
6. Leamon, C.P.; Reddy, J.A.; Vlahov, I.R.; Westrick, E.; Parker, N.; Nicoson, J.S.; Vetzel, M. Comparative preclinical activity of the folate-targeted vinca alkaloid conjugates EC140 and EC145. *Int. J. Cancer* **2007**, *121*, 1585–1592.
7. Müller, C.; Struthers, H.; Winiger, C.; Zhernosekov, K.; Schibli, R. DOTA conjugate with an albumin-binding entity enables the first folic acid-targeted ^{177}Lu-radionuclide tumor therapy in mice. *J. Nucl. Med* **2013**, *54*, 124–131.
8. Selhub, J.; Rosenberg, I.H. Demonstration of high-affinity folate binding activity associated with brush border membranes of rat kidney. *PNAS* **1978**, *75*, 3090–3093.
